# Trial of Multidisciplinary Observation at an Expandable Sub-Marine Cabled Station “Off-Hatsushima Island Observatory” in Sagami Bay, Japan

**DOI:** 10.3390/s91109241

**Published:** 2009-11-18

**Authors:** Takafumi Kasaya, Kyohiko Mitsuzawa, Tada-nori Goto, Ryoichi Iwase, Keizo Sayanagi, Eiichiro Araki, Kenichi Asakawa, Hitoshi Mikada, Tomoki Watanabe, Ichiro Takahashi, Toshiyasu Nagao

**Affiliations:** 1 Japan Agency for Marine–Earth Science and Technology (JAMSTEC), 2-15, Natsushima-cho, Yokosuka, Kanagawa, 237-0061, Japan; E-Mails: mitsuzawak@jamstec.go.jp (K.M.); tgoto@tansa.kumst.kyoto-u.ac.jp (T.G.); iwaser@jamstec.go.jp (R.I.); araki@jamstec.go.jp (E.A.); asakawa@jamstec.go.jp (K.A.); 2 Institute of Oceanic Research and Development, Tokai University, 3-20-1, Orido, Shimizu-ku, Shizuoka-shi, 424-8610, Japan; E-Mail: sayanagi@scc.u-tokai.ac.jp (K.S.); nagao@scc.u-tokai.ac.jp (T.N.); 3 Department of Civil and Earth Resources Engineering, Graduate School of Engineering, Kyoto University, Kyoto daigaku-Katsura, Nishikyo-ku, Kyoto, 615-8540, Japan; E-Mail: mikada@tansa.kumst.kyoto-u.ac.jp (H.M.); 4 Marine Works Japan Ltd. / c/o JAMSEC YES, 3173-25, Syowa-machi, Kanazawa-ku, Yokohama, Kanagawa, 236-0001, Japan; E-Mails: tomwat@jamstec.go.jp (T.W.); itaka@jamstec.go.jp (I.T.)

**Keywords:** multidisciplinary observation, expandable submarine cabled station, ocean bottom electro-magnetometer

## Abstract

Sagami Bay is an active tectonic area in Japan. In 1993, a real-time deep sea floor observatory was deployed at 1,175 m depth about 7 km off Hatsushima Island, Sagami Bay to monitor seismic activities and other geophysical phenomena. Video cameras monitored biological activities associated with tectonic activities. The observation system was renovated completely in 2000. An ocean bottom electromagnetic meter (OBEM), an ocean bottom differential pressure gauge (DPG) system, and an ocean bottom gravity meter (OBG) were installed January 2005; operations began in February of that year. An earthquake (M5.4) in April 2006, generated a submarine landslide that reached the Hatsushima Observatory, moving some sensors. The video camera took movies of mudflows; OBEM and other sensors detected distinctive changes occurring with the mudflow. Although the DPG and OBG were recovered in January 2008, the OBEM continues to obtain data.

## 1. Introduction to the Hatsushima Observatory

**Figure 1.**
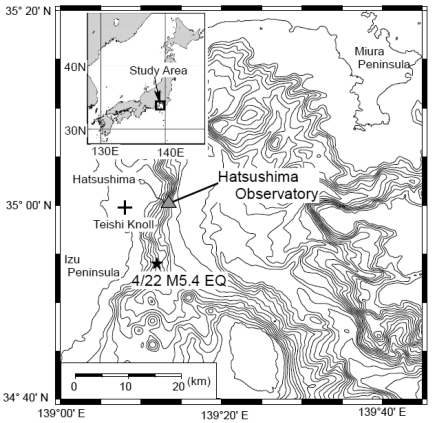
Location of the Hatsushima Observatory. A star shows the epicenter of the off-Izu Peninsula earthquake that occurred on 22 April 2006. Teishi Knoll is a submarine volcano that erupted in 1989.

Sagami Bay is a plate subduction zone along the Sagami Trough in Japan. It is an active tectonic area (Figure 1). Earthquake activity is very high in the western part of Sagami Bay, east of the Izu Peninsula. Submarine volcanoes have erupted repeatedly. For example, Teishi Knoll erupted in 1989 (Figure 1). Moreover, biological research has been conducted in this area and individual biological colonies have been discovered.

**Figure 2.**
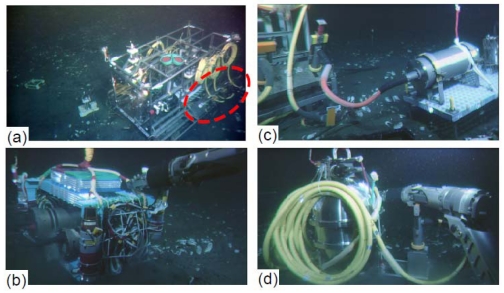
(a) Photograph of the renewed Hatsushima Observatory. Red dashed line shows serial ports. (b) An ocean bottom electromagnetometer set up near the Hatsushima Observatory by the ROV Hyper Dolphin. (c) An ocean bottom differential pressure gauge (DPG) system connected to the serial port. (d) Photograph of an ocean bottom gravimeter during emplacement operation.

For multidisciplinary observation to monitor geophysical and biological phenomena, the Japan Agency for Marine–Earth Science and Technology (JAMSTEC) planned to construct a submarine observatory at a depth of 1,175 m about 7 km off Hatsushima Island in Sagami bay. The original observatory system was deployed and set up in 1993 [1]. This observatory had a seismometer, hydrophone, thermometer, a conductivity, temperature, and depth instrumentation (CTD), video camera, and an electromagnetic current meter. However, it had no submarine external port. Use of the original system was halted in 1999 because of a submarine cable fault; it was recovered in 2000. The system was completely renovated in 2000 for carrying out more multi-disciplinary observations with various other sensors, and for development of a more expandable system with underwater mateable connectors, aside from the reason that the primary observatory had broken [2]. Figure 2 (a) portrays a photograph of the renovated underwater observatory system (35.003083N, 139.2247E).

The new system is equipped with a transmissometer, an Acoustic Doppler Current Profiler (ADCP), a tsunami pressure gauge (a precision pressure gauge), and a gamma ray spectrometer, as well as sensors of the same kind used in the previous configuration of the observatory. The performance of some of those sensors has since been improved. The A/D sampling of seismometer is 24 bits/200 Hz. One of the two video cameras is a High-gain Avalanche Rushing Photo conductor (SuperHARP) camera, which is far more sensitive than a typical CCD camera [3]. Table 1 presents specifications of the renovated Hatsushima system. It is remarkable that it has four underwater mateable connectors for additional observation instruments (Figure 2). Various data obtained on the sea floor have been sent to Yokohama Institute, JAMSTEC, through the Hatsushima land station, and stored on a data server. Registered users can preview the time series data using a web browser, and download data from the data center server (http://www.jamstec.go.jp/scdc/top_e.html). This web site has provided other observatory data: data of the off-Kushiro–Tokachi and off-Muroto cable observatories installed by JAMSTEC for real-time earthquake and tsunami disaster-prevention. The off-Kushiro–Tokachi system, which was detailed in an earlier report [4], observed various remarkable phenomena such as tsunami generation processes, turbidity current, etc. during the 2003 Tokachi-oki earthquake of M8 [5]. In this paper, we explain the operations by which we added some observation systems to the Hatsushima system. We also report some interesting data obtained using the observatory equipment when a large earthquake occurred.

## 2. Deployment of New Additional Equipment at the Hatsushima Observatory and Time Series

**Figure 3.**
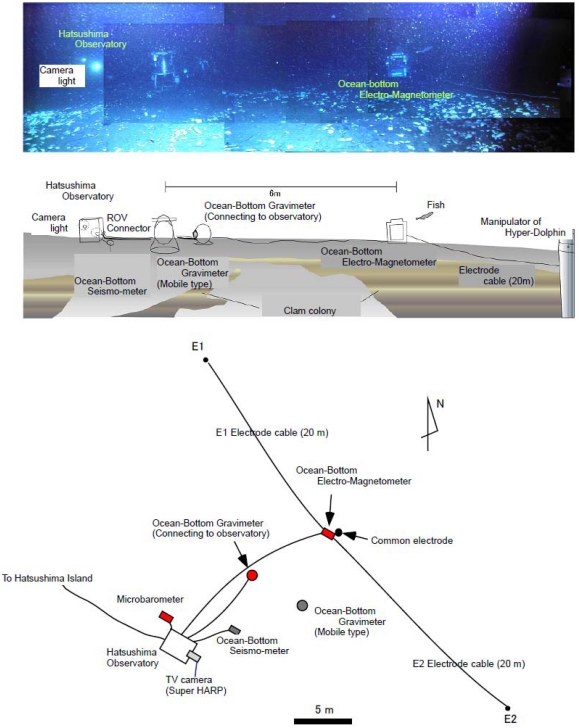
(Upper panel) Photograph and arrangement map of various instruments. (Lower panel) Plain view around the Hatsushima Observatory. Red marks show instruments placed by Hyper-Dolphin.

During February 2005, we connected an ocean bottom electro-magnetometer (OBEM), an ocean bottom differential pressure gauge system (DPG) and an ocean bottom gravimeter (OBG) to the Real-Time Deep Sea Floor Observatory at 1175 m depth offshore of the Hatsushima Island in Sagami Bay using the Research Vessel “Natsushima” and the Remotely Operated Vehicle “Hyper-Dolphin” during the NT05-01 Cruise. We then initiated long-term, real-time observation tests on the ocean bottom (Figure 2). These instruments were laid out within a radius of about 10 m and were connected to underwater mateable connectors (Figure 3). Table 2 presents the specifications of each added measurement system.

**Figure 4.**
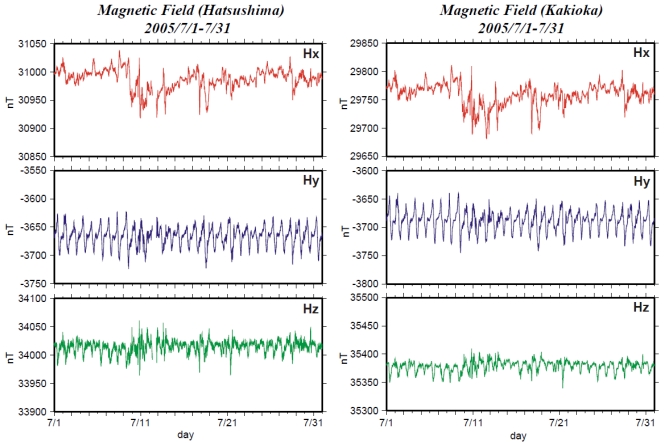
Comparison of the magnetic component's monthly variation deduced by Hatsushima Observatory and Kakioka Observatory in Ibaragi prefecture.

**Figure 5.**
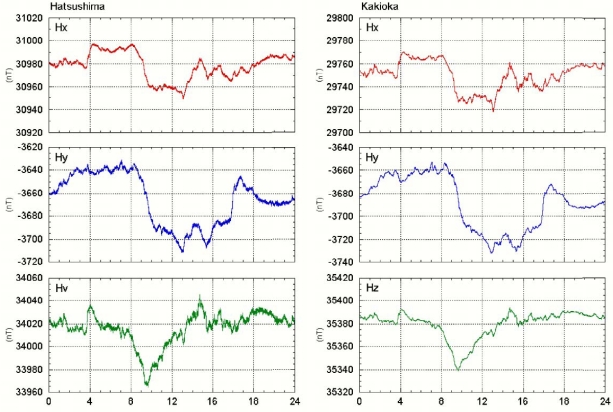
1-day comparison of the magnetic component's daily variation, as deduced by Hatsushima Observatory and Kakioka Observatory in Ibaragi prefecture.

Tokai University developed the OBEM system and performed tests in shallow sea [6]. This system comprises three components: (1) fluxgate magnetometer and inclinometer, (2) an electric potentiometer and (3) a main CPU unit. Electrical power is supplied from the observatory. The main CPU unit controls data measurements and communications. All sensors and electrical units are installed in pressure cases made of aluminum alloy mounted in an aluminum frame (Figure 2). This system can measure three magnetic field components, two horizontal electric field components and two inclinometer components (pitch and roll angle) with a sampling rate of 1, 2, 4 or 8 Hz. Figures 4 and 5 portray comparisons of magnetic field time series of the Hatsushima Observatory and the Kakioka geomagnetic observatory (36.2322N, 140.1864E). The coordinate system of magnetic measurement is rotated to true north. Figure 4 shows the monthly time variation during July 2005. The daily variations were observed very clearly; both time variations are very similar. The one-day variation on 21 July 2005 is presented in Figure 5. Our OBEM data include higher frequency variations than those presented in Kakioka's data.

**Figure 6.**
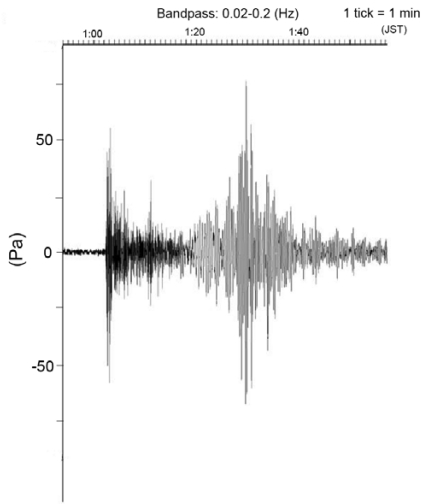
Clear surface waves as well as P and S phases from the Sumatra Island offshore earthquake on 29 March 2005, as sensed by this DPG at the seafloor observatory.

We also installed a differential pressure gauge (DPG) system and an ocean bottom gravimeter (OBG) system. The DPG system is based on that described by Cox *et al.* [7] to monitor long-period pressure changes such as low-frequency earthquakes and teleseismic earthquakes. As a result of the system calibration, this sensor's response is smaller at frequencies lower than 0.059 Hz. Figure 6 portrays clear surface waves as well as P and S phases from a large earthquake offshore of Sumatra Island observed using this DPG in the seafloor observatory. The DPG system was recovered to improve the system performance. We reconnected the improved DPG system to the Hatsushima station in January 2008 again. The improved DPG system includes sensors such as a strong motion accelerometer and a quartz pressure gauge to expand its capability to monitor seismic and pressure signals.

An OBG system consists of a gravimeter (SB-3M; Scintrex Ltd.), a pressure meter (Paroscientific Inc.), a sealed backup battery, and a CPU module for communication and telemetry with each sensor and land station. The sensors, with gimbals and a communication unit, were enclosed in a titanium sphere. Watanabe *et al.* [8] reported that the OBG system has 0.07 mGal/day linear drift and the residual gravity data with linear drift removed shows very long-period fluctuation with amplitude of more than 2 mGal. The OBG system was recovered during the NT08-01 cruise. We will analyze those data.

## 3. Time Variations Caused by the Mudflow During the Off-Izu Earthquake

**Figure 7.**
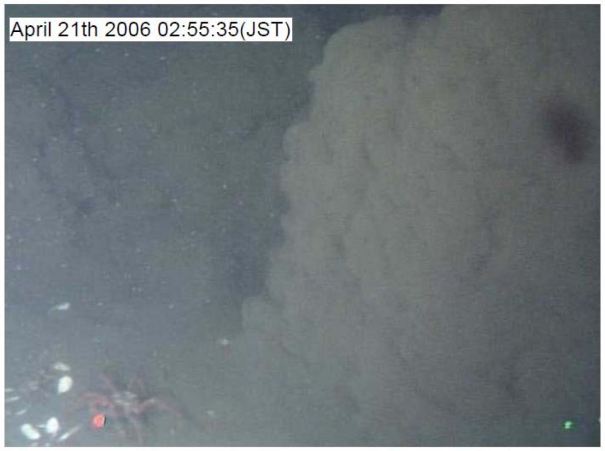
Captured image of the mudflow at 2:55 taken using the SuperHARP camera.

**Figure 8.**
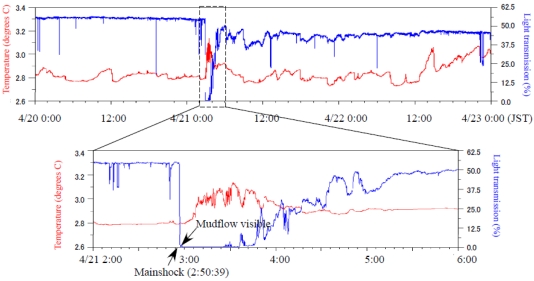
Temperature and light transmission record before and after the off-Izu Peninsula earthquake. The lower panel shows detailed waveforms around the mudflow arrival.

**Figure 9.**
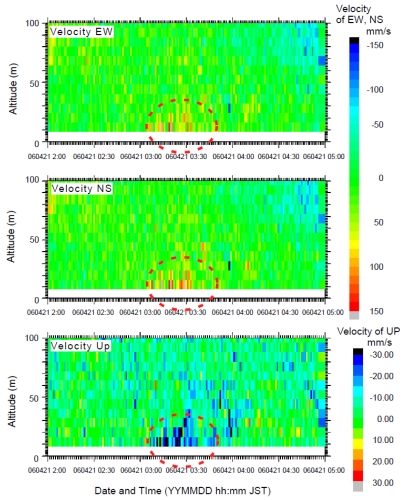
Three components of the current velocity time variations obtained using the ADCP sensor. The current direction changed downward and northeastward at 3:10. The mudflow thickness was estimated as about 30 m.

A large earthquake (M5.4) occurred off-Izu Peninsula at 2:50:39 on 21 April 2006, generating a submarine landslide. The generated mudflow reached the station about five minutes after the seismic wave arrived. Kinoshita *et al.* [9] reported that some sensor positions were shifted by the mudflow; the SuperHARP camera took a movie of the mudflow at 2:55 (Figure 7). Figure 8 presents temperature and light transmission data before and after the earthquake occurrence. After the toe of the mudflow main body reached the observatory, the light transmission ratio became almost zero. The low light transmission ratio continued for at least 2 hrs. The temperature showed only a small change when the mudflow toe reached the observatory. However, a substantial change was detected after 3:10. Figure 9 presents current velocity time variations obtained using an ADCP sensor. The current direction changed downward northeast at 3:10. This change might have occurred as the main body of the mudflow reached the observatory. Moreover, the maximum velocity change of each component showed at a height of about 30 m at 3:30. Therefore, this mudflow thickness was estimated as about 30 m.

**Figure 10.**
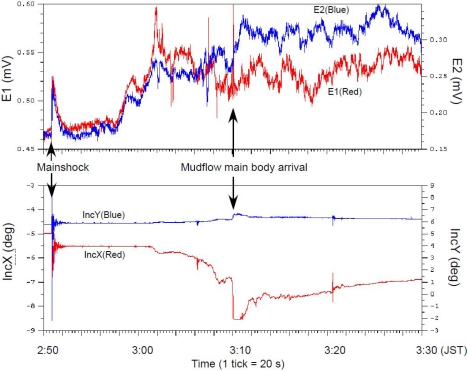
Time variations deduced by OBEM after the earthquake. The upper panel shows two electrical components; the lower panel shows inclinometer variations.

Figure 10 depicts the OBEM data obtained before and after the occurrence of the earthquake and mudflow. Magnetic components and inclinometer data showed, at first, high-frequency changes resembling those seen on the seismometer. A small data offset existed because the OBEM was shaken as the seismic body waves arrived. Electrical components also changed suddenly. However, these sudden variations differed from the variation of a magnetic sensor and inclinometer. Both electronic components rose, then reverted to the original data trend with the same phase. Subsequently, the inclinometer and electrical components started a gradual change before the mudflow main body reached it. Both electrical components increased at 2:58 just immediately after light transmission data became almost zero (Figure 8). Inclinometers showed gradual changes occurring simultaneously with the lack of visibility. Finally, large step-like changes occurred after the mudflow main body's arrival; then the waveform decayed. The magnetic components showed similar changes simultaneously. Furthermore, each electrical component diverged and showed a negative correlation after the arrival of the mudflow's main body.

## 4. Discussion

We succeeded in the connection of some new geophysical equipment at the Hatsushima Observatory. Moreover, we obtained data related to some interesting features associated with a large earthquake that occurred near our observatory. Salient features were elucidated using some sensors at the time of the mudflow occurrence. The SuperHARP camera and light transmission data recorded the arrival of the mudflow toe at 2:55 (Figures 7 and 9). However the variances of other sensors were not great. Subsequently, the electric field and the inclination of OBEM and the temperature started indicating anomalous behaviors (Figures 7–10). These features are interpreted as the first arrival of the initial mudflow. Finally, they showed a substantial change at around 3:10. Furthermore, the ADCP sensor also detected the strong current flow. These geophysical phenomena, which were expected to have been caused by the growing mudflow, were first discovered by these multidisciplinary sensors, and will be used for discussion of the mudflow's initiation, growth and progress.

The electric field variation caused by the mudflow is especially interesting. In general, the movement of electrically conductive seawater and geomagnetic field can generate an electric field on the seafloor [10]. The correlation among the electric field, the inclination of OBEM and the current velocity by ADCP implies that the gradual electrical change will result from the motionally induced electric field according to the gradual growth and movement of the mudflow. Results show that each electrical component (Figure 10) started varying with a positive correlation before the arrival of the mudflow main body. Then, they diverged and sometimes showed a negative correlation after the arrival of the mudflow main body. Because two electric fields are parallel with the NW–SE direction but have mutually opposite sign each other (see Figure 3), a negative correlation will be caused by a far electric source generating a uniform electric filed around the OBEM. Moreover, a positive correlation or less correlation will be interpreted as a localized electric field because of a source near the OBEM electrodes. Therefore, the observed electric field associated with the mudflow can be possibly explained by turbulent flow with both small-scale (near the OBEM) and large-scale (far from the OBEM) water currents. Further analyses of data from OBEM and other sensors will enable us to clarify the detailed growth process of the mudflow.

Another interesting variation is the anomalous behavior of electric field immediately after the seismic wave arrival (at 2:51–53, Figure 10). This variation including its decay (about 40 s) is too early to be explained by the mudflow. Therefore, another source for the electric field is need to explain the anomalous change. One candidate for the source is the fluid flow below the seafloor related with shaking by s seismic body wave. The electrokinetic effect associated with the movement of pore fluid in rocks and sediments can create observable electrical potential on the ground surface [11], which is called the streaming potential. Possible evidence of fluid seepage from the seafloor is presented by a seafloor geochemical monitoring at the time of this event. Gamo *et al.* [12] reported an abrupt manganese anomaly related to earthquake occurrence using a submersible automatic manganese analyzer, which is called GAMOS and which was also connected with the Hatsushima observatory before the earthquake. They concluded that a manganese change results from the sporadic fluid supply from seafloor sediments through a local path near the Hatsushima observatory, formed by the earthquake. The motion of pore water will produce a localized streaming potential. Actually, the two components of observed electric field at the arrival of seismic wave showed a positive correlation. As discussed above, this phenomenon might results from a localized electric source around the common electrode, which is consistent with the sporadic seepage at the earthquake proposed by Gamo *et al.*[12]. These multidisciplinary observations possibly related to fluid seepage at the earthquake provide opportunities for quantitative analysis of fluid seepage and seafloor liquefaction by shaking, which will be attempted in the near future.

## 5. Conclusions

We report the connection of some new geophysical equipment at the Hatsushima Observatory. We have presented an overview of the capabilities of the Hatsushima Observatory for multidisciplinary observations using the submarine cable. It is noteworthy that the OBEM system has obtained electromagnetic data continuously for over four years. Moreover, data from some equipment showed some interesting features related to a large earthquake that occurred near the observatory. Salient features were elucidated using the OBEM. The electric field variation related with the fluid flow is an especially interesting phenomenon.

Our success has yielded much experience from the construction of the multidisciplinary observation. Our OBEM system, in particular, has been gathering electro-magnetic data since January 2005, with observations of salient results for the cabled observatory. Making use of these experiences, we have been constructing a new observatory system off the Tokai region [13,14]. For analyses to yield more precise results, we plan to improve the observatory system further.

## Figures and Tables

**Table 1.** Specifications of the “off-Hatsushima system”.

**Seismometer**	Three component servo velocimeter (Manufacturer : Tokyo Sokushin Co., Ltd.)
	Range: 1 m/s FS (Low gain), 1 cm/s FS (High gain)
	24 bit/200 Hz sampling

**Hydrophone**	Model : ITC-1010A (Omni-directional)
	Receive sensitivity : −183 dB/V/uPa
	24 bit/200 Hz sampling

**TV camera**	SuperHARP camera (Model : OVS-SHK-506A) × 1
	Sensitivity : 130 Lux/F : 2.0
	3CCD camera (Model : OVS-152) × 1
	Sensitivity : 2,000 Lux/F : 8.0

**CTD**	Model : SeaBird SBE-9/17plus with Light transmissometer (Model : ALPHA TRACKA2)
	Range : Conductivity : 0–7 S/m,
	Temperature : −5 to 35 °C,
	Pressure: 0 to 2,000 psi
	Transmissometer : 0 to 100% (@ 660 nm)
	Sampling interval : 1 sec.

**Sub-bottom thermometer**	Thermistor type thermometer (Manufacturer : Nichiyu Giken Kogyo Co., Ltd.)
	4ch probe × 2
	Range : −10 to 50 °C
	Sampling interval : 10 s.

**ADCP**	Model : RD Instruments BB-DR-150
	Range : Current velocity : <10 m/s
	Altitude : 12 to 484 m / 8 m interval
	Sampling interval : 1 min

**Current meter**	Model : Sontec ADVOcean acoustic current meter
	Range : 1 mm/s−5 m/s
	Sampling interval : 10 s

**Gamma ray spectrometer**	3 inch spherical NaI(Tl) scintillator (Manufacturer : Shonan Co., Ltd.)
	Number of channels : 256
	Sampling interval : 1 min

**Tsunami pressure gauge**	Model : Paroscientific 8B2000-I
	Range : 0–20 MPa
	Sampling interval : 10 s.

**Underwater light**	Halogen lamp (95 V/250W) × 6

**Power supply**	DC 840 V
**Mateable connector**	19.2 kbps serial connectors
	RS-232, 15V/1A DC power supply × 3
	RS-422, 15V/2.4A DC power supply × 1
	Optical connectors × 4

**Submarine cable**	Double armoured electro-optical cable
	Electrical line × 4; Optical line × 12

**Table 2.** Specifications of the OBEM, DPG and OBE.

**OBEM Specifications**	

Magnetic sensor	
Sensor type	Fluxgate
Resolution	0.01 nT
Components	X, Y and Z
Dynamic Range	327.67 nT
Electoric Potentiometer	
Number of component	2 components
Sensor span	20 m
Inclimeter	
Resolution	0.001 deg
Control unit	
Sampling rate	1, 2, 4 and 8 Hz selectable
Communication port	RS-232C

**DPG Specifications**	

Senstivity	1550 count /Pa
Frequency range	10 mHz - 5Hz
Sampling rate	10 Hz
A/D convertor	24 bit
Noise level(1Hz-5Hz)	5 Pa rms
Max. pressure	7,000 Pa

**OBG Specifications**	

Resolution	1 μGal
Obs. Range	700 mGal
Accuracy	5 μGal
Direction and inclinometer	
Direction accuracy	0.5 deg(RMS)
Direction resolution	0.1 deg
Inclinometer accuracy	0.2 deg
Inclinometer resolution	0.1 deg
Inclinometer range	20 deg
